# Supplementary material for: Functional Analysis of GSTK1 in Peroxisomal Redox Homeostasis in HEK-293 Cells
Source: Antioxidants (Basel). 2023 Jun 7;12(6):1236. doi: 10.3390/antiox12061236 (PMC10295636; doi:10.3390/antiox12061236)
Supplement: Supplementary file 1 [file antioxidants-12-01236-s001.zip › Costa et al_Legends to Supplementary Figures_R1_unmarked version.pdf]

antioxidants

Legends to Supplementary Figures

Figure S1. Immunoblot quantification of three biological replicates for the experiment depicted in **Figure 2 and 3A**. The band intensities were normalized to ACTB, and the relative protein expression (fold change) was obtained by dividing the intensity of each protein band by the average band intensity of the corresponding protein in the control group. The means are represented by horizontal lines. The data obtained for the CT and ΔGSTK1 cells were statistically compared by two-way ANOVA with Tukey's multiple comparison test (*, $p < 0.05$). There were no statistically significant differences between Cl1 and Cl2.

Figure S2. Representative images of HEK-293 cells expressing the fluorescent redox sensors indicated. CT and ΔGSTK1 (cl 1 and 2) HEK-293 cells were electroporated with a plasmid encoding cytosolic (c-) or peroxisomal (po-) versions of roGFP2, roGFP2-Orp1, SoNar, cpYFP, mKeima, iNAP1, or iNAPc and grown in rMEMα medium for two to three days before imaging. Scale bar, 10 µm.

Figure S3. Responsiveness of po-roGFP2 to various oxidative insults. CT or ΔGSTK1 (cl 1 or 2) HEK-293 cells were transfected with a plasmid encoding (**A**) c-roGFP2-Orp1 or (**B-D**) po-roGFP2. (**A,B**) The cells were first cultured for three days in rMEMα containing 1 µg/mL doxycycline and 500 nM Shield1 (to express and stabilize DD-DAO, respectively) and subsequently chased for one day in the same medium lacking doxycycline and Shield1 (to degrade the residual cytosolic pool of DD-DAO that has not yet been imported into peroxisomes). Next, the cells were incubated in DPBS supplemented (+) or not (-) with D-alanine (D-Ala), and the F400/F480 response ratios of (**A**) c-roGFP2-Orp1 ($n = 1$; 9 or 10 individual measurements per time point) and (**B**) po-roGFP2 ($n = 2$; 20 individual measurements per time point) were monitored over time. (**C**) The cells were cultured for two days in rMEMα and the basal oxidation state (b) of po-roGFP2 was measured. Next, the cells were exposed for 10 min to 400 µM H_2O_2 ($n = 3$; 30 individual measurements per time point) and the F400/F480 response ratios of po-roGFP2 were measured again at the indicated time points. (**A-C**) All response ratios were normalized to the average value of the corresponding basal condition. The data points and vertical bars represent the mean and standard deviation of the individual measurements. For each time point, the data acquired for the ΔGSTK1 conditions were statistically compared with those obtained for the CT cells using the two-way ANOVA test with Tukey's multiple comparisons test, but no significant differences were found. (**D-E**) The cells were cultured for two days in rMEMα. Next, the cells were exposed for 10 min to the indicated concentrations of diamide or AT-4 and the F400/F480 response ratios of po-roGFP2 were measured. The data are presented as violin plots (the horizontal solid and dashed lines denote the median and the first and third quartiles, respectively) and were statistically compared with the non-treated groups using the Kruskal-Wallis test with Dunn's multiple comparisons test (*, $p < 0.05$; **, $p < 0.01$; ***, $p < 0.001$).
