# Supplementary figures and images for: Functional Analysis of GSTK1 in Peroxisomal Redox Homeostasis in HEK-293 Cells
Source: Antioxidants (Basel). 2023 Jun 7;12(6):1236. doi: 10.3390/antiox12061236 (PMC10295636; doi:10.3390/antiox12061236)

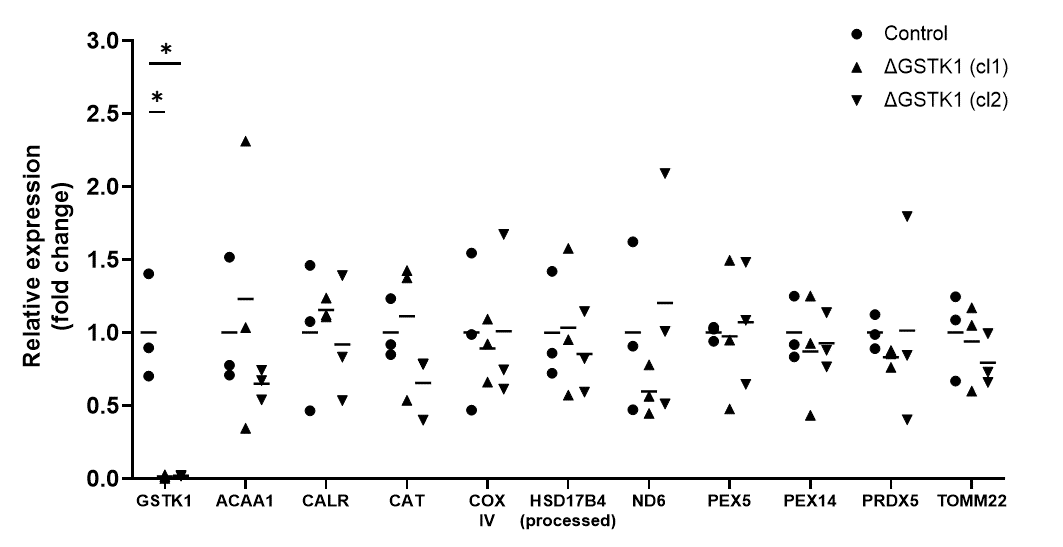

Supplement: Supplementary file 1 [file antioxidants-12-01236-s001.zip › Figure S1.png]

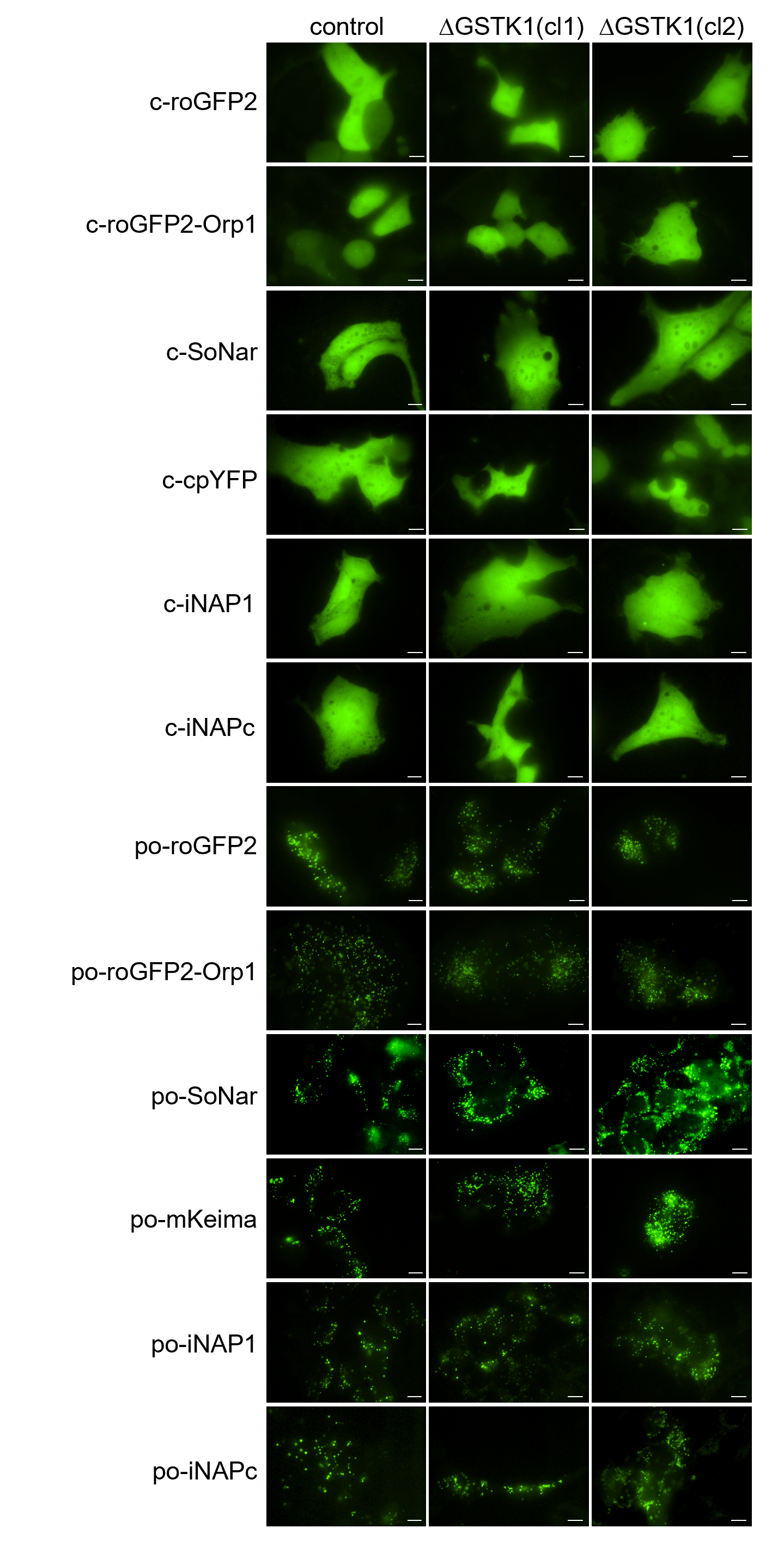

Supplement: Supplementary file 1 [file antioxidants-12-01236-s001.zip › Figure S2.png]

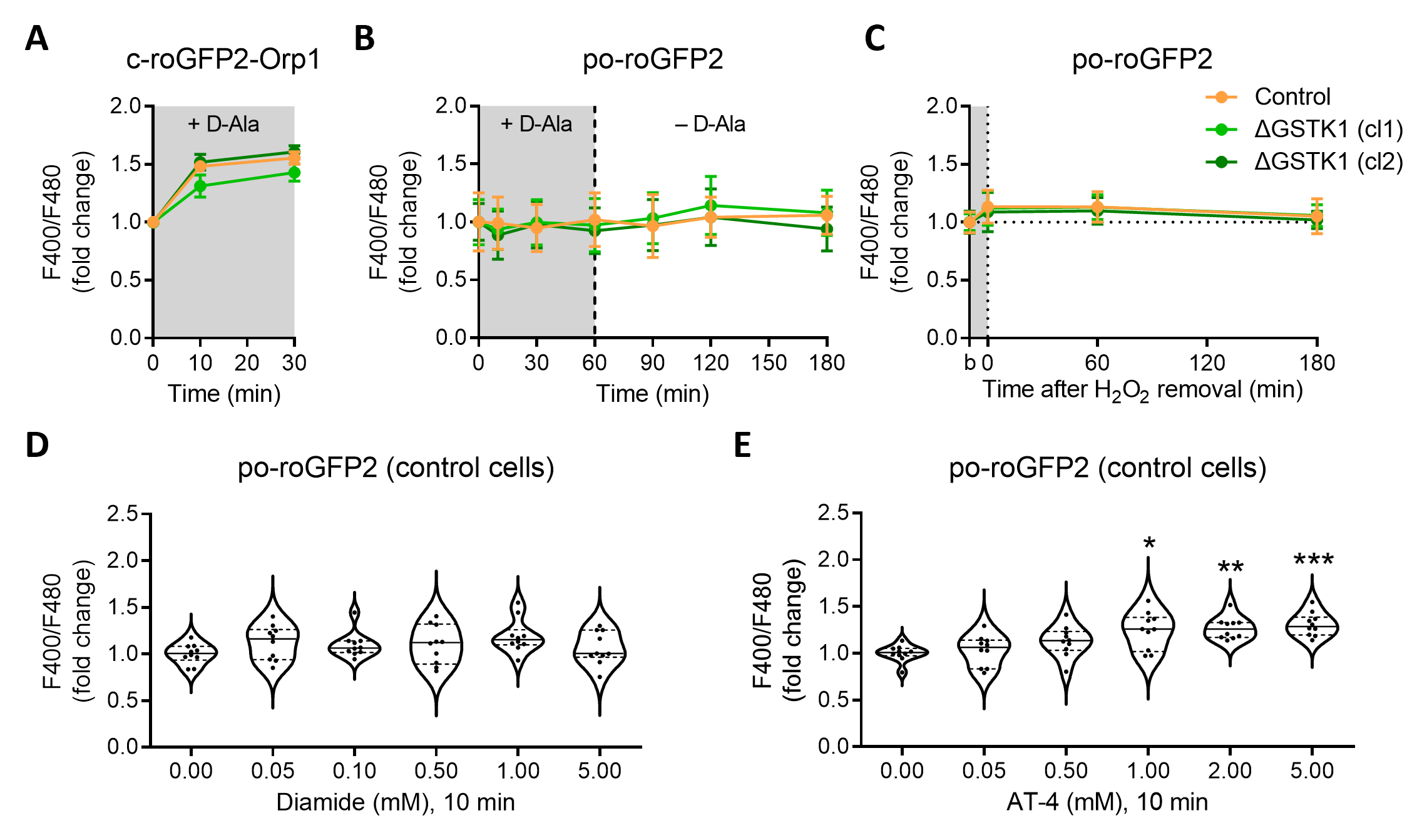

Supplement: Supplementary file 1 [file antioxidants-12-01236-s001.zip › Figure S3.png]
